# Clinical Society of Maryland

**Published:** 1892-06

**Authors:** W. T. Watson

**Affiliations:** Sec’y; 1603 N. Broadway, Baltimore, Md.


### Society Notes.

### CLINICAL SOCIETY OF MARYLAND.

BALTIMORE, March 4, 1892.

The 263rd regular meeting was called to order by the President, Dr. Robert W. Johnson.

DR. HERBERT HARLAN spoke of a case of buphthalmus, which had recently come under his care. Buphthalmus is simply a very large eye. It is very rare; in over 26,000 cases at the Presbyterian Eye and Ear Hospital, not one case of buphthalmus has been recorded. There are some cases put down as bulging of the cornea and probably one or two of them were cases of congenital buphthalmus. Dr. Harlan's case is a girl of 11 years; both eyes effected, but one larger than the other. The condition was noticed at birth; eyes gradually grew worse. Sight was never good In one eye the cornea became completely opaque and there was a good deal of inflammation and pain for six months, and then all light perception was lost. The other eye has likewise been somewhat painful; it is enormously enlarged, almost as big as the eye of an ox. Cornea of about usual thickness; tension about normal; iris a little thin and apparently a little stretched about margin. Eye exceedingly myopic. It looked like any other eye except its enormous size. Quite a number of cases of enlarged eyes are reported, but they are not perfect like this one. Dr. Harlan saw another similar case recently in a negro man, but the eye was defective. It was 1 1-2–1 3-4 inches in diameter. The eye was removed. Vitreous and lens opaque, cornea cloudy and ciliary region inflamed.

DR. ROBERT RANDOLPH: I found about a year ago, in a bottle at the Eye and Ear Hospital, a bulpthalmic eye. It was evidently taken from a child 6 or 8 years ago. There was a marked cylinder-shaped excavation of the optic nerve very much as we see in glaucoma. The eye was thin; it was full of holes showing that it was much atrophied. The ciliary body was atrophied; cornea cloudy.

I think Nettleship reports a case in which iridectomy has done good. I understand that there is a case in France where the vitreous is systematically drained.

DR. CHAMBERS: Was the rest of the face symmetrical in Dr. Harlan's case?

DR. HARLAN: She is a very pretty little girl.

DR. RANDOLPH: Is it not generally one-sided?

DR. HARLAN: It was double in both cases of mine, but one eye larger than the other. In these two cases, I looked at the optic nerve and there was no depression. I think it likely that the one Dr. Randolph found was one of secondary glaucoma.

DR. RANDOLPH: It was one of hydro-opthalmus in a child.

DR. J. L. INGLE reported a case of osteomyelitis, in which the symptoms during life were so misleading that the real nature of the disease was only discovered at the autopsy. A healthy bcy of 14 was for several days less vivacious than usual, then on Nov. 20th, '91, was taken seriously sick. Previous to the 20th, there had been a festered spot on left ankle which had apparently healed. On 20th, fever 104 deg., severe pain in left leg from knee to toes. Fever high till 22d, when it fell to normal. On this day had some epistaxis and on following day tympanites marked. Acute rheumatism was diagnosed, and salicylate of soda given. Mind wandered from the first, constant headache from beginning to end with persistent insomnia. On the 23d, Dr. Earle suspected typhoid fever from delirium and epistaxis. Dr. Ingle was inclined to regard it as a case of cerebral meningitis; the child lay with both eyes closed, was irritable when aroused, avoided light, was constipated, surface pallid, erythematous blushing, dilated pupils. Instead of a retracted abdomen there was an enormously distended one. At this time there was marked soreness all over the body. Patient put upon iodine and bromide of soda, but no benefit derived. Tympanitis increased and interfered with respiration and circulation.

The child died, and an autopsy was made by Dr. Chambers.

DR. J. W. CHAMBERS: The meninges of brain were markedly congested and there was just such a condition as one would expect as the result of sepsis. There had been, seemingly, no definite cause for sepis. The sore on the leg had scabbed over and was dry. Looking over the extremity it was observed that his left leg over the tibia was enlarged and œdematous, and this caused us to cut down. The periosteum was œdematous and red and easily detached. When ripped away quite a large amount of bloody pus oozed from the center of the tibia. No other bones were allowed to be examined, although from the fact that he had had pain in the other leg and elsewhere in the body, we suspected that it was a case of multiple osteomyelitis involving several bones.

As an idiopathic disease, osteomyelitis has not received much attention until within the past few years. The cause of the disease is probably simply the pus microbe getting into this particular locality. It is probably always secondary to some pus formation elsewhere in the body. Some think it is absorbed from the alimentary tract, the bronchial mucous membrane or other sources and then finds its way into the the bones—mostly young bones near the epiphyses. The disease is often difficult to diagnose. Holmes has said that average number of these cases are diagnosed on the autopsy table. We have few definite symptoms to guide us. I do not think any doctor should feel chagrined if he should fail to diagnose osteomyelitis in such a case as the present one. It is probable that many cases die under our care that we have diagnosed as something else. I have seen but three cases, two I recognized at once, the third only after the death of the child. The only distinctive symptom, was intense pain at one point. By pressing over the bone, if the individual is not thoroughly affected by the septic condition, you can find tenderness. The prognosis is bad; multiple cases are very little modified by medical or surgical treatment. Where only one bone is involved, if we recognize it or suspect it, I should not hesitate to trephine. We can in this way save a number of lives or a good deal of destruction of tissue. I would not have any more hesitation in boring into a pus cavity in bone than in opening an abscess in the soft tissue. In multiple cases the sepsis is so great that there is scarcely anything to be done either by surgeon or physician. Under these circumstances I would not hesitate to give relief to tensions.

DR. W. S. THAYER: I saw, some 3 years ago, at the Massachusetts General Hospital, a case very similar to the one reported, except that the bone was the femur. A boy of 14 was admitted to the medical side with a diagnosis of sciatica. He had been treated by a reputable physician for ten days with this diagnosis. For a week before entrance, had had occasional chilly sensations, but no actual chill. He was in a typhoid condition, dry, brown tongue and high fever. I found upon examination that the upper part of the thigh on one side was distinctly larger than that on the other side, somewhat tense to feel and rather hot. He was immediately transferred to the surgical side, an opening was made and a large quantity of pus was found. The boy was in a state of septicæmia from which he did not recover.

DR. S. T. EARLE reviewed the literature of osteomyelitis with especial reference to causation.

DR. J. M. T. FINNEY: This disease in a certain number of cases is difficult to diagnose; in certain other cases it is not. I happen to have seen two or three of the latter class. As far as the pathology is concerned, I think it is simply a bone abscess. The treatment is like the treatment of a like condition in other tissues, viz., where there is pus evacuate it.

The resemblance of this disease to appendicitis is very marked. It gets well in many cases and will return again. As we are coming more and more to operate in appendicitis, so we ought more and more to operate in myelitis.

There is one point in diagnosis which Dr. Chambers has not referred to and which seems to me a most important one, viz., local œdema.

Local œdema seems to me to be the most characteristic sign of pus deeply seated.

Dr. Finney related 3 cases operated upon by himself, all making good recovery. The staphylococcus pyogenes aureus was found present in all these cases.

DR. CHAMBERS: As to œdema, a good many cases may die from sepsis before œdema is present. If you get pain and œdema then the diagnosis is more sure.

DR. S. T. EARLE: I saw the case reported in the early stage. There was no point of localized tenderness. He was tender from the knee down, with no point worse than another. In about two days after the onset there was just as much tenderness over the right limb as over the left, and this hyperæsthetic condition soon became general. As to œdema, the limb was only a trifle larger in the whole extent from the knee.

DR. J. M. T. FINNEY then read an exhaustive paper on "Appendicitis."

DR. W. S. THAYER: A number of men in Munich have collected 1000 cases of appendicitis in the Munich hospitals. A German doctor has analyzed these cases, and arrives at the conclusion that appendicitis is fully as common in females as in males, if, indeed, it is not more common. As to age, he finds the proportion almost exactly the same in old age as in youth.

Operation must be governed by surroundings. In a large city where we have good surgical skill, I believe that where the symptoms progress 24 hours the case should be handed over to the surgeon. I believe the majority of these cases belong to the surgeon.

DR. J. D. BLAKE: There is no doubt but that under proper conditions an early operation is advisable. To induce the patient's family to permit the operation to be done at his home or at a hospital is often impossible.

I was rather struck with Dr. Finney's idea of combining all these conditions—typhlitis, perityphlitis and appendicitis—because it is a difficult thing to know just which you have.

I remember that Dr. Chew once said that at a meeting of the American Medical Association the physicians were discussing appendicitis on the medical side, and concluded that at a very early day such cases should be handed over to the surgeon for operation. At the same time the surgical section were discussing the same thing, and concluded that an operation should not be performed too early; that it was better to wait.

I have seen two cases where an early operation showed that the trouble was located in the appendix, which was removed. In two other cases that I have seen there was so much adhesion that it was difficult to determine where the trouble was.

I remember one case, in a young man, where the aspirator was used to determine the presence of pus. Three and one-half ounces of pus were drawn. It was thought better not to withdraw it all, as the walls of the abscess might collapse, and there would be a tear into the abdominal cavity. The patient has since had no further trouble. Eight months ago I was called to a young man with appendicitis. I advised operation, which was declined. Five days later I aspirated him, and drew away nearly four ounces of pus. The next day I drew away a little over two ounces of pus. This patient has never had any trouble since. I had a similar case six months ago, in which I aspirated twice. He has since complained of pus about that region, and I have recommended an operation. Certainly where the diagnosis is not plain, an aspirator rarely jeopardizes the case, and very often throws some light upon the trouble. In another case in which I used an aspirator I got about three ounces of blood, and the symptoms all disappeared.

DR. J. F. MARTINET: I am distinctly a medical man, but I have been converted through personal experience to the belief that appendicitis is distinctly a surgical trouble. The cases which I have attended have come on abruptly with acute pain. I have had four cases in my practice within twelve months. Two of the cases Dr. Chambers saw with me. An operation was advised, but in neither of the cases would the family consent. My habit at present in every case I meet with is to state that it is a distinctly surgical trouble and refer it to the surgeon. One of my cases who refused operation secured relief by suppuration through the bowels. This was a year ago. In July last she passed through another attack safely. Lately she was operated upon by Prof. Kelly and is now well.

DR. J. W. CHAMBERS: The statistics from the medical side seem to be faulty. A person may have three or four attacks, and they are reported as four cases cured. A surgeon operates upon a case and reports one case.

As to women having it as frequently as men, I have under my own observation known of three women upon whom the gynæcologists had diagnosed pelvic cellulitis and salpingitis, and removed the "tube" which proved to be the appendix. In a large number of cases the appendix is really a pelvic organ, and if inflamed it will certainly be a case of salpingitis with some gynæcologists. Such errors may have something to do with statistics. There is no reason in the world why women should escape more than men; certainly they are just as liable to catarrhal affections.

Referring to aspiration, I think Dr. Blake's patients are relieved rather than cured. I should hesitate to use the needle. If I should find pus with the aspirator, I should never feel that I had done my duty unless I had cut down and removed all the pus. It would not be good treatment for an abscess in the iliac region.

DR. INGLE: I believe the sooner we place these cases in the hands of the surgeon, the better. When the family would not consent to an operation I have treated these cases with salines and enamata without any relief whatever.

DR. THAYER: In the Munich statistics, it is said that undoubtedly cases of appendicitis had been called salpingitis and pelvic cellulitis, and many cases in the female were doubtless missed in this way.

DR. MARTINET: Two of my cases occurred in females, one of 5 years, the other of 10 years of age.

DR. BLAKE: There are certain cases where I can find no distinct indication of what my patient has, whether it is typhlitis, perityphlitis or appendicitis, and when I have waited long enough, I put in an aspirator to find out if there is pus and in no case have I seen bad symptoms follow, and in some there was distinct benefit. I think we are thoroughly justified in using an aspirator to make a diagnosis.

DR. FINNEY: As these are pus cases, the operation can be done as well at the patient's home as in a hospital.

As to aspiration, I agree entirely with Dr. Chambers. Where there are sufficient indications for aspiration, there are still more indications for the use of the knife.

DR. W. T. WATSON, Sec'y.
1603 N. Broadway, Baltimore, Md.

